# Native mass spectrometry enabled by infrared matrix-assisted laser desorption electrospray ionization for rapid measurement of protein-ligand biophysical parameters

**DOI:** 10.1007/s00216-025-06229-9

**Published:** 2025-11-14

**Authors:** Adeleke A. Adepoju, Reza A. Ghiladi, David C. Muddiman

**Affiliations:** 1 Biological Imaging Laboratory for Disease and Exposure Research (BILDER), Department of Chemistry, North Carolina State University, Raleigh, NC 27695 USA; 2 Department of Chemistry, North Carolina State University, Raleigh, NC 27695 USA

**Keywords:** Protein-ligand complex, Dissociation constant, Binding affinity, Biophysics, IR-MALDESI

## Abstract

The determination of an equilibrium dissociation constant (*K*_d_) alongside the maximum binding capacity (*B*_max_) of protein-ligand interactions is essential in understanding binding affinities and binding capacities which play a major role in the design and development of new therapeutic molecules. Rapid and accurate determination of these biophysical parameters (*K*_d_ and *B*_max_) in noncovalent protein-ligand interactions is desirable in the modern drug discovery process. Previously, we demonstrated the detection of noncovalent protein-ligand complexes by infrared-assisted matrix desorption electrospray ionization-mass spectrometry (IR-MALDESI-MS). Here, we report the first determination by IR-MALDESI-MS of biophysical parameters from noncovalent protein-ligand interactions using a ligand titration approach. Unlike conventional electrospray ionization-mass spectrometry (ESI-MS), IR-MALDESI-MS enables analysis in < 13 s per ligand concentration emphasizing its speed, automation, and sensitivity. Native mass spectrometry by IR-MALDESI was performed on carbonic anhydrase II (CAH) incubated with sulfanilamide (SLFA), a known inhibitor. Coupled with other published studies, these results demonstrate that IR-MALDESI has strong potential as a high-throughput screening (HTS) technique for rapidly and accurately determining the *K*_d_ and *B*_max_ of protein-ligand complexes.

**Graphical abstract:**

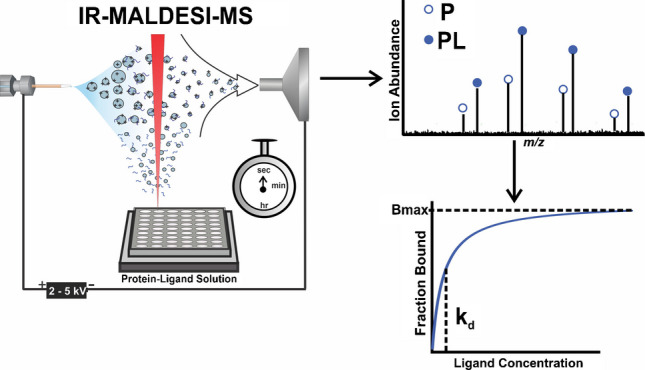

**Supplementary Information:**

The online version contains supplementary material available at 10.1007/s00216-025-06229-9.

## Introduction

Accurate characterization of noncovalent protein-ligand interactions and their associated biophysical parameters including stoichiometry, *K*_d_, and *B*_max_ is key to the drug discovery process [1, 2]. The significance of this characterization has led to the use of multiple analytical methods such as mass spectrometry [2–4], surface plasmon resonance (SPR) [5–7], isothermal titration (ITC) [8–10], fluorescence polarization [11], and nuclear magnetic resonance (NMR) [12–15] to determine their values. These values provide insights into how small molecules engage their targets at the molecular level, thereby providing in-depth biological understanding and guiding the design of more effective therapeutics [16]. The pathogenesis of various diseases has been linked to protein-mediated biological processes. As such, the inhibitory mechanism of some small molecules targeting these proteins has facilitated the drug discovery process by competitively binding to the enzyme substrate active site through covalent or noncovalent bonds [1, 16]. Inhibitory mechanisms mediated through noncovalent interactions are driven by a range of forces including van der Waals forces, hydrogen bonding, electrostatic interactions, and hydrophobic forces. While covalent interactions offer prolonged biological effects, dynamic biological processes are desirable in the biopharmaceutical field, thereby making noncovalent inhibitors valuable for their reversible and selective modes of action [1].

Mass spectrometry (MS) is widely used as an analytical technique for the analysis of various biomolecules [1, 17], and when coupled with different ionization techniques such as electrospray ionization (ESI) [18–22], desorption electrospray ionization (DESI) [23], liquid extraction surface analysis nano-electrospray ionization (LESA-nESI) [2], and matrix-assisted laser desorption/ionization (MALDI) [24, 25]. It has been applied to quantify noncovalent protein-ligand interactions by directly detecting and quantifying the gas phase ions of ligand-bound and unbound protein. IR-MALDESI is a hybrid and soft ionization source that combines the benefits of ESI and MALDI [26, 27], and has been successfully applied in the detection of noncovalent ligand complexes [1]. Its compatibility with automated workflows enables high-throughput (HT) analysis, offering a distinct advantage over conventional ESI-MS [27–29]. Moreover, IR-MALDESI has been established as a platform for the HTS of small molecules in the initial stages of drug discovery, achieving a sampling rate of 22 wells per second [30–33], and for *K*_d_ determination, it offers a superior data acquisition speed (seconds) when compared to other analytical techniques [1, 34].

Due to the rate of emerging diseases, the need for the rapid development of novel drug candidates is increasingly critical [35]. Following hit identification in the drug discovery process, the accurate and rapid measurement of biophysical parameters for compounds that bind to their target(s) becomes essential during the optimization process. Investigating the binding affinity and binding capacity of large numbers of small molecules can be time consuming, pointing to a need for a HT approach that can provide reliable biophysical data within a reasonable time.

In this study, we harnessed the potential of IR-MALDESI and reported the first demonstration of its application in native mass spectrometry to measure the biophysical parameters of noncovalent protein-ligand interactions. As a proof of concept for the technique, we employed a previously well-characterized system of carbonic anhydrase II (CAH) with sulfanilamide (SLFA), a known noncovalent binder. At a fixed concentration of CAH, SLFA was titrated, and the resulting ion abundance for each charge state was used for *K*_d_ and *B*_max_ determination. This study demonstrates the potential of IR-MALDESI-MS as an analytical platform for the rapid and accurate determination of *K*_d_ and *B*_max_ of noncovalent protein-ligand interactions, thereby establishing the groundwork for future studies.

## Experimental section

### Sample preparation

CAH II (C3934, ≥ 2000 W-A units/mg, Sigma-Aldrich, Allentown, PA USA) was prepared at a concentration of 10 µM in 100 mM ammonium acetate buffer (pH ~ 7.0, Sigma-Aldrich, Burlington, MA, USA). A 100-µL aliquot of the protein stock solution was desalted using a 7-kDa molecular weight cutoff Zeba spin desalting column (89833, Thermo Fisher Scientific, San Jose, CA, USA) which was pre-equilibrated with 20 mM ammonium acetate buffer (pH ~ 7.0). A 3 mg/mL stock solution of SLFA (Selleck Chemicals, Houston, TX, USA) was prepared in dimethyl sulfoxide (DMSO, Sigma-Aldrich, WI, USA) and subsequently diluted with water to yield a 400-µM working solution. While the CAH was at a fixed concentration, varying concentrations of the SLFA (0, 3, 5, 10, 20, 40, 60, 80, 100, 160, 200, and 250 µM) were prepared, added to the protein solution, and adjusted to volume with 20 mM ammonium acetate. The mixtures were incubated at room temperature for 15 min to facilitate binding. For each SLFA concentration, 30 µL of the incubated mixture was transferred into a 35-µL well plate (384, MilliporeSigma, Burlington, MA, USA) for analysis by IR-MALDESI-MS.

### IR-MALDESI-MS analysis

IR-MALDESI studies using a well plate were performed as previously described [1]. Briefly, the incubated mixture was pipetted into the wells of a 384 well plate and placed on the three-dimensional (3D) stage and IR-MALDESI control software (RastirZ) enabled movement of the stage for HTS [28]. Ionization polarity was set to positive mode with an electrospray solvent composition of 20 mM ammonium acetate in 50% methanol (Fisher Scientific, Nazareth, PA, USA), and a flowrate of 1.5 µL/min with 3700 V applied to the conductive union of the electrospray emitter to form a stable orthogonal spray plume. Ammonium acetate was used in the electrospray solvent composition to preserve the native state of CAH during MS analysis. A mid-IR-laser (JGM Associates, Inc., Burlington, MA, USA) which was set up to fire 10 pulses per burst (PPB) with an energy of 1.4 mJ per burst was focused on the surface of the sample solution in the wells, and fired to abate neutral sample droplets which get ionized in an ESI manner. The multi-RF threshold was set to 4, automatic gain control (AGC) was turned off to allow a fixed optimized injection time of 125 ms [36], and the mass spectra were collected between mass-to-charge ratios (*m*/*z*) of 1000–4000 with a resolving power of 15,000 full-width half-max (fwhm) at *m*/*z* 200 to enable distinct separation of individual charge states.

### Data analysis

For each ligand concentration, 25 mass spectra were acquired at a sampling rate of 2 Hz, giving a total acquisition time of approximately 12.5 s. The raw spectra were averaged and viewed using Freestyle software (Thermo Fisher Scientific, v1.3 SP2). Ion abundances corresponding to free and ligand-bound protein were extracted for each charge state using the spectrum list within the software workspace, and the fraction of protein bound was calculated as the ratio of bound protein to the total protein (bound + unbound) (Table S1, Equations S1 and S2). Fraction-bound values were plotted against free ligand concentrations (Table S2), and the parameters *K*_d_ and *B*_max_ were derived by nonlinear regression of the titration curve using a one site-specific binding model in GraphPad Prism (GraphPad Software v10.5.0, San Diego, CA, USA).

## Results and discussion

**Fig. 1:**
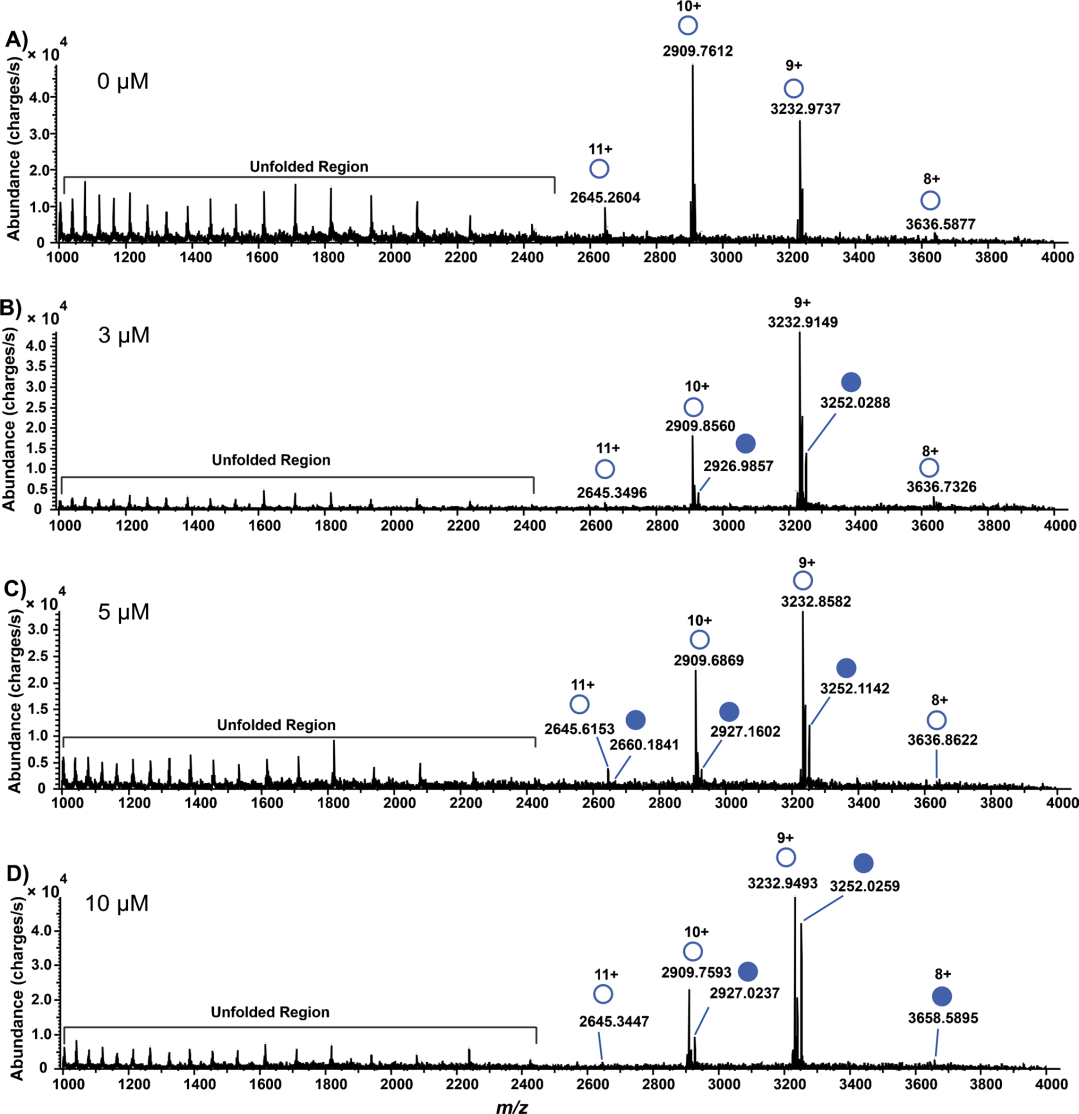
Native mass spectra of CAH with different concentrations of SLFA at the ligand depletion stage with 10 µM desalted protein plus **A** 0 µM SLFA; **B** 3 µM SLFA; **C** 5 µM SLFA; and **D** 10 µM SLFA. The filled circles represent the protein-ligand-bound complex reflecting a resultant mass shift upon binding, while the empty circles represent free CAH. Unfolded regions represent denatured protein

CAH is a metalloenzyme that has a well-defined active site containing a single zinc ion that is essential for ligand coordination and catalysis [37]. Figure 1A shows the mass spectra of desalted CAH without SLFA, and Fig. 1B–D shows various points of the titration with SLFA.

The charge state envelope ranges from 11+ to 8+. Denaturation was observed in the unfolded region. In addition to chemical and physical factors that can cause protein denaturation, the protein’s own internal dynamics and inherent marginal stability may contribute to its denaturation [38–41]. To mitigate this during analysis, CAH was dissolved in ammonium acetate buffer which was also incorporated in the ESI solvent to preserve its native state and minimize denaturation. However, low abundant peaks were still detected in the unfolded region of the mass spectrum, indicating that a fraction of the total CAH was denatured during analysis. Comparing the spectra from pure CAH to when it has been incubated with SLFA, a reduction in the peak abundance of the unfolded region is observed. This observation is consistent with a ligand-induced stabilization mechanism, whereby SLFA binding to the zinc-containing active site likely locks CAH in a more stable folded conformation, thereby shifting the conformational equilibrium toward its native state [42–45].

**Fig. 2:**
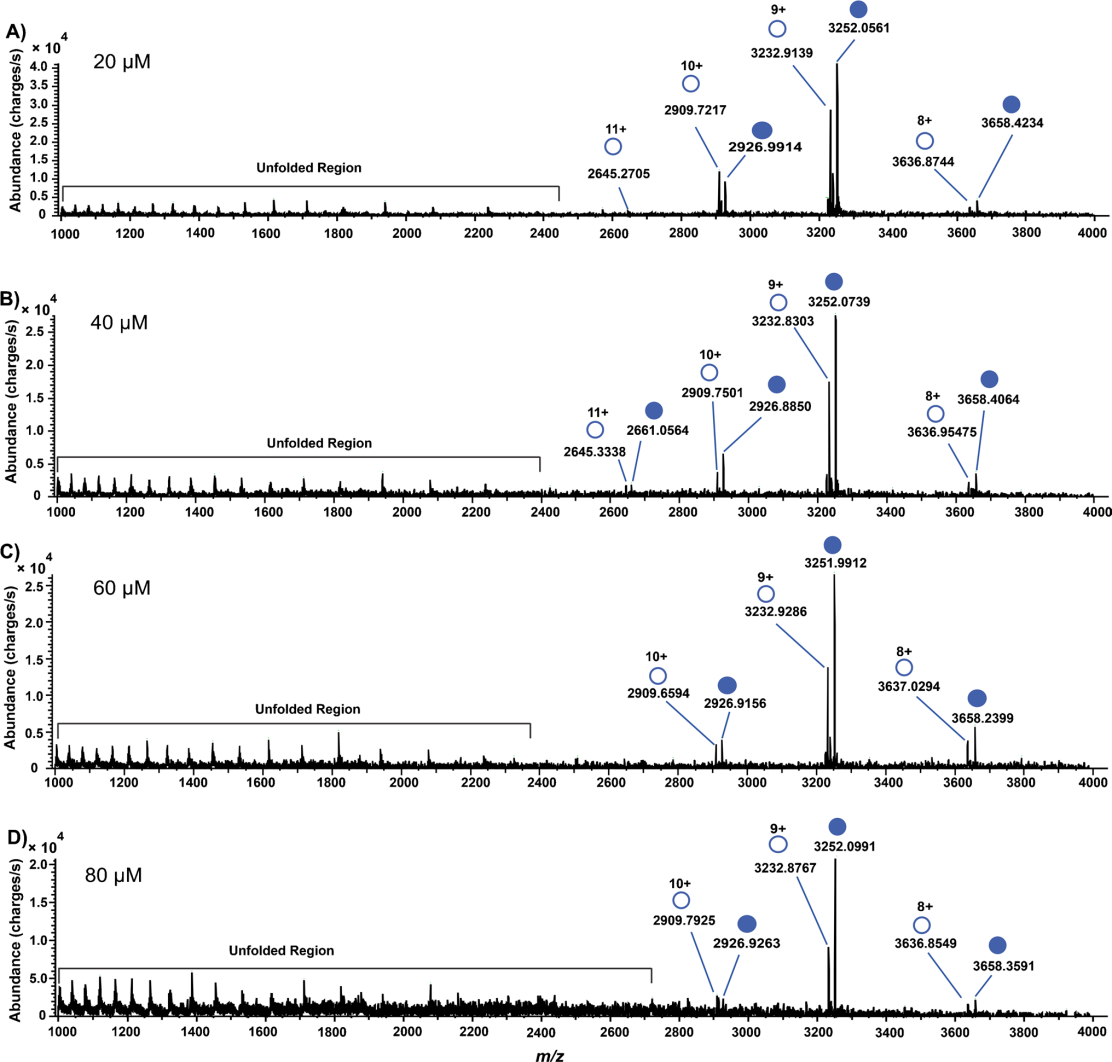
Native mass spectra of 10 µM CAH with [SLFA] > [CAH]. **A** 20 µM SLFA; **B** 40 µM SLFA; **C** 60 µM SLFA; and **D** 80 µM SLFA. The filled circles represent the protein-ligand-bound complex showing a resultant mass shift upon binding, while the empty circles represent free CAH. Unfolded regions represent denatured protein

**Fig. 3:**
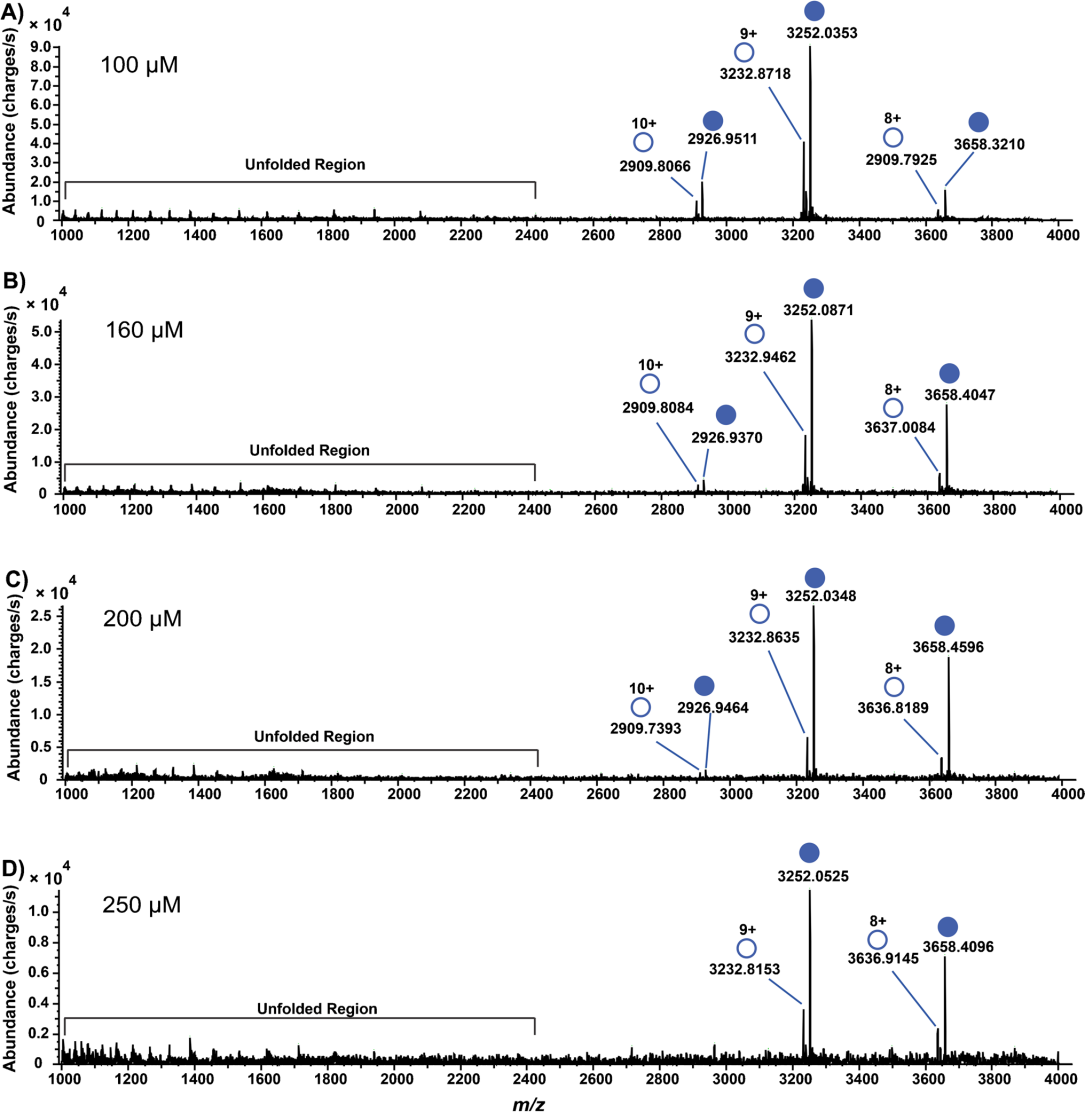
Native mass spectra of 10 µM CAH with concentrations of [SLFA] > [CAH]. **A** 100 µM SLFA; **B** 160 µM SLFA; **C** 200 µM SLFA; and **D** 250 µM SLFA. At these SLFA concentrations, CAH becomes almost saturated. The filled circles represent protein-ligand-bound complexes reflecting a resultant mass shift upon binding, while the empty circles represent free CAH. Unfolded regions represent denatured protein

Shown in Figs. 2 and 3 are the mass spectra acquired under native MS conditions where the SLFA concentration is higher than that of the CAH. Relative to the unbound or free CAH, progressive increases in SLFA concentration resulted in increased ion abundance for the CAH-SLFA complex, accompanied by a shift in charge state distribution at a higher SLFA concentration.

**Fig. 4:**
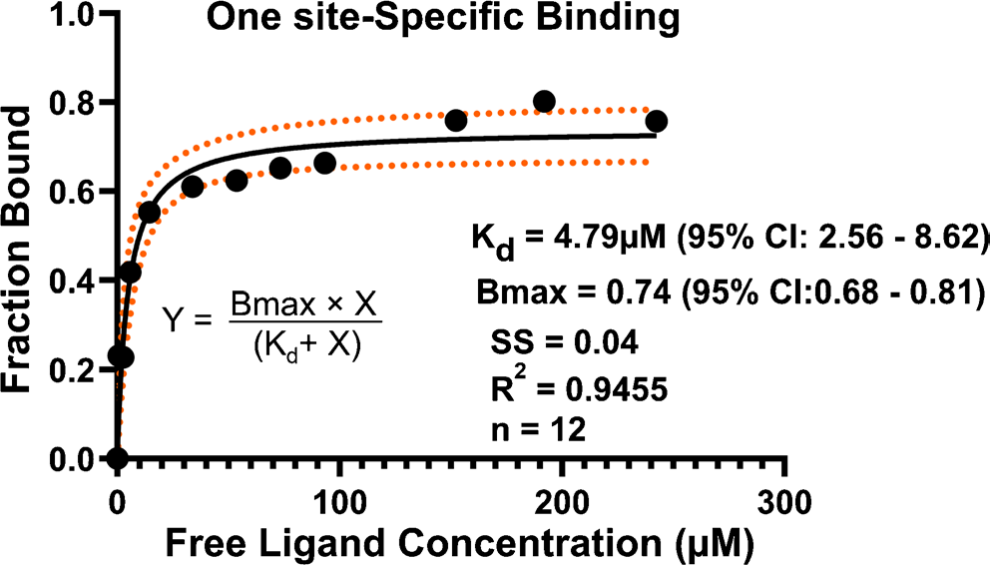
A nonlinear regression curve showing the *K*_d_ and *B*_max_ of the CAH-SLFA noncovalent interaction and other statistical parameters. The fraction of bound CAH was plotted against free SLFA concentrations and fit to a one site-specific binding model. *Y* and *X* denote the fraction bound and the free ligand concentrations, respectively. The solid black line represents the best-fit curve, the red dotted lines denote the 95% confidence interval (CI), SS denotes the sum of squares, *n* denotes the number of points, and *R*^2^ denotes the coefficient of determination

As seen in Fig. 2C through Fig. 3C, a charge state envelope contraction from four observable charge states (+11 to +8) to three (+10 to +8) was observed. This charge contraction was first observed at 60 μM of SLFA. At 250 μM of SLFA (Fig. 3D), the charge distribution is narrowed down to two dominant charge states (+9 to +8). This phenomenon can be explained by the IR-MALDESI mechanism, where laser ablation liberates the CAH-SLFA mixture into the gas phase, and subsequent interaction with the orthogonal electrospray plume leads to protonation of the CAH basic groups such as lysine, arginine, histidine and the N-terminus, which are the charge carriers. This contraction of charge states is also expected for any platform that ionizes in an ESI manner. At high SLFA concentration, native CAH becomes more stable, and the exposure of the charge carriers becomes reduced, thus limiting the number of available protonation sites and leading to fewer protons transferred during ionization. This reduction in charge states reflects ligand-induced stabilization and a decrease in Coulombic repulsion between protonated sites, enhancing the stability of the compact protein-ligand complex in the gas phase [46, 47]. Although 10 µM of CAH was used in this analysis, IR-MALDESI has been reported to detect purified protein solutions at concentrations as low as 10 pM [26]. Biophysical parameters obtained in this study using a one site-specific binding model include the *K*_d_ and *B*_max_. The nonlinear regression curve with its binding model and other statistical parameters is presented in Fig. 4.

To account for SLFA depletion, the fraction of bound protein was plotted against the free ligand rather than the total ligand concentration. Minor differences in free SLFA values were observed at higher SLFA total concentrations compared to lower total concentrations, where ligand depletion is more pronounced. Details of the data analysis and free SLFA calculation equation are provided in the electronic Supplementary Material (Tables S1-S2, Equation S1-S4). The equation for the 1:1 binding model used is denoted on the plot and yields a *K*_d_ of 4.79 µM (95% CI, 2.56–8.62 µM). The red dotted lines in the plot indicate the 95% CI, representing the range of possible curves around the best-fit curve shown in black. The measured *K*_d_ indicates a moderate noncovalent interaction between CAH and SLFA. As expected, our IR-MALDESI MS result agrees with the *K*_d_ values reported in the literature for SLFA binding to CAH by LESA-MS (3.2 µM ± 1.68 [2]) and SPR (4.42 µM ± 1.39 [2], 3.1 µM ± 1.10 [48] and 5.88 µM ± 0.06 [49]); however, it is important to note that our experimental sampling time is less than 13 s, versus tens of minutes to hours for determination by SPR [50, 51]. While MALDI-TOF and Echo MS using acoustic ejection mass spectrometry (AEMS) achieve faster acquisition speed [52, 53], the differences reflect the fundamental mechanism of the Orbitrap mass analyzer used in this study, which relies on transient-based detection [54]. The MS data acquisition parameters used were previously optimized for HTS and native IR-MALDESI analysis. These MS settings are needed to set the balance between acquisition speed and spectral quality [36]. Importantly, the CAH-SLFA biophysical parameters obtained are consistent with literature values from both secondary and MS-based methods, supporting the reliability and validity of this study. Saturation was observed with a *B*_max_ of 0.74 (95% CI, 0.68 to 0.81), which is lower than the theoretical maximum of 1. We attribute this to two possible reasons: First, the denatured protein observed in the unfolded region in the mass spectra, even though it appears to have a low abundance, could potentially decrease the total number of binding sites, thereby resulting in a lower *B*_max_ value. Second, the commercially available CAH used in this study was reported to have an activity of 2000 W/A units/mg, which is less than the maximum reported activity of 3500 W/A units/mg [1]. A lower activity could result in a reduction of available binding sites, thus decreasing the observed *B*_max_. This interpretation aligns with observations by Illes-Toth et al., [2] who reported a *B*_max_ of 0.63 using a CAH preparation of unspecified activity, further emphasizing that protein quality and its preparation could potentially affect binding capacity. Importantly, while *B*_max_ reflects the fraction of active binding sites, the *K*_d_ remains unchanged, as it represents the intrinsic affinity of SLFA for native CAH. Nonlinear regression provided an excellent fit to the binding data, with a sum of squares of 0.04 and a coefficient of determination (*R*^2^) of 0.946.

## Conclusion

The IR-MALDESI platform allows for the detection of noncovalent protein-ligand interactions and associated parameters (*K*_d_ and *B*_max_). This study positions IR-MALDESI at the forefront of rapid and accurate determination of biophysical parameters for noncovalent interactions, owing to its softness, specificity, and high-throughput capabilities. While this platform has been adopted in the biopharmaceutical industry, this concept would improve the lead optimization process during drug discovery. We are currently optimizing conditions for stronger binders and plan to extend this study to nanomolar affinity noncovalent interactions.

## Supplementary Information

Below is the link to the electronic supplementary material.

Supplementary Material 1 (DOCX 27.1 KB)

## Data Availability

The data that is contained in this study is available in NSCU’s Dryad Dataset: 10.5061/dryad.tht76hfcb.
