# Supplementary material for: Native mass spectrometry enabled by infrared matrix-assisted laser desorption electrospray ionization for rapid measurement of protein-ligand biophysical parameters
Source: Anal Bioanal Chem. 2025 Nov 14;418(3):775–82. doi: 10.1007/s00216-025-06229-9 (PMC12890989; doi:10.1007/s00216-025-06229-9)
Supplement: Supplementary file 1 — Supplementary Material 1 (DOCX 27.1 KB) [file 216_2025_6229_MOESM1_ESM.docx]

**Native Mass Spectrometry Enabled by Infrared Matrix-Assisted Laser Desorption Electrospray Ionization for Rapid Measurement of Protein-Ligand Biophysical Parameters**

Adeleke A. Adepoju^1^, Reza A. Ghiladi^2^, David C. Muddiman^1^*

*^1^Biological Imaging Laboratory for Disease and Exposure Research (BILDER), Department of Chemistry, North Carolina State University, Raleigh, NC 27695, USA*

*^2^ Department of Chemistry, North Carolina State University, Raleigh, NC 27695, USA*

**Supporting Information**

**2 Tables and 4 Equations**

**Submitted to:** *Analytical and Bioanalytical Chemistry*

**Submitted:** September 27, 2025

Revised: October 30, 2025

**Keywords:** Protein-Ligand Complex, Dissociation constant, Binding affinity, Biophysical parameters, IR-MALDESI

***Author for Correspondence**

David C. Muddiman, Ph.D.

Biological Imaging Laboratory for Diseases and Exposure Research (BILDER)

North Carolina State University

Phone: 919-513-0084

Email: [dcmuddim@ncsu.edu](mailto:dcmuddim@ncsu.edu)

**Table of Contents**

**Table S1:**  Details of the abundance values extracted for different concentrations of

sulfanilamide

**Table S2:** Calculated values for fractional bound and free SLFA concentration

**Equation S1:** Equation to express ligand depletion at low SLFA concentrations

**Equation S2:**  Equation to show how CAH fraction bound is calculated using the ion abundance of

free CAH and bounded complex

**Equation S3** Equation showing how the concentration of CAH-SFLA complex is calculated to

accurately account for free SLFA

**Equation S4:**  Equation expressing how free SFLA is calculated.

**Table S1:** Extracted ion abundance for each bounded and unbounded charge state. Red represents abundance for unbounded charge state and blue represents abundance of bounded charge state.

| **Total SFLA Concentration (µM)** | **2645** | **2660** |  | **2909** | **2926** |  | **3232** | **3252** |  | **3636** | **3658** |
| --- | --- | --- | --- | --- | --- | --- | --- | --- | --- | --- | --- |
|  |  |  |  |  |  |  |  |  |  |  |  |
| 0 | 0 | 0 |  | 4259.83 | 0 |  | 6817.49 | 0 |  | 0 | 0 |
| 3 | 1703.44 | 752 |  | 18351.2 | 4092.91 |  | 43876.57 | 13880.69 |  | 3289.51 | 1520.8 |
| 5 | 3943.96 | 1281.75 |  | 22366.04 | 3735.83 |  | 33818.05 | 12288.02 |  | 1207.58 | 811 |
| 10 | 1763.65 | 775.48 |  | 22,960.00 | 9188.03 |  | 49,746.70 | 42141.57 |  | 1789.75 | 2603.32 |
| 20 | 1169.73 | 0 |  | 11953.14 | 9262.46 |  | 28774.25 | 41232.9 |  | 2303.09 | 4164.36 |
| 40 | 1665.42 | 1773.34 |  | 3717 | 6529.47 |  | 17562.47 | 27597.05 |  | 2247.65 | 3529.34 |
| 60 | 1244.87 | 653.51 |  | 3309.18 | 3892.44 |  | 13831.49 | 26601.52 |  | 3788.62 | 5572.13 |
| 80 | 0 | 0 |  | 2753.81 | 2314.86 |  | 9223.05 | 20924.98 |  | 1573.19 | 2171.04 |
| 100 | 0 | 0 |  | 1011986 | 1981303 |  | 41099.11 | 90636.74 |  | 5477.2 | 16012.18 |
| 160 | 0 | 0 |  | 2756.33 | 4222.26 |  | 18113.98 | 53759.63 |  | 6403.75 | 27770.3 |
| 200 | 0 | 0 |  | 1161.96 | 1434.35 |  | 6610.68 | 26101.67 |  | 3663.38 | 18762.14 |
| 250 | 0 | 0 |  | 0 | 0 |  | 3607.03 | 11438.72 |  | 2345.52 | 7152.23 |

**Table S2:** Calculated values used to accurately determine the CAH fraction bound and free SLFA concentration. The CAH bound fraction is the plotted against the FREE SLFA concentration.

| **Sum of all CAH bounded and free abundance** |  | **Sum of CAH bound abundance** |  | **CAH fraction bound** |  | **Complex Concentration [PL] (µM)** |  | **Free SLFA concentration (µM)** |
| --- | --- | --- | --- | --- | --- | --- | --- | --- |
|  |  |  |  |  |  |  |  |  |
| 11077.32 |  | 0 |  | 0 |  | 0.00 |  | 0.00 |
| 87467.12 |  | 20246.4 |  | 0.231474 |  | 2.31 |  | 0.69 |
| 79452.23 |  | 18116.6 |  | 0.228019 |  | 2.28 |  | 2.72 |
| 130968.5 |  | 54708.4 |  | 0.417722 |  | 4.18 |  | 5.82 |
| 98859.93 |  | 54659.72 |  | 0.552901 |  | 5.53 |  | 14.47 |
| 64621.74 |  | 39429.2 |  | 0.610154 |  | 6.10 |  | 33.90 |
| 58893.76 |  | 36719.6 |  | 0.623489 |  | 6.23 |  | 53.77 |
| 38960.93 |  | 25410.88 |  | 0.652214 |  | 6.52 |  | 73.48 |
| 3146514 |  | 2087952 |  | 0.663576 |  | 6.64 |  | 93.36 |
| 113026.3 |  | 85752.19 |  | 0.758693 |  | 7.59 |  | 152.41 |
| 57734.18 |  | 46298.16 |  | 0.801919 |  | 8.02 |  | 191.98 |
| 24543.5 |  | 18590.95 |  | 0.757469 |  | 7.57 |  | 242.43 |

**Equation S1:** Accounting for ligand depletion

[L] _free_ ≠ [L] _total_ when ligand concentration is lower than protein concentration.

**Equation S2:** Fraction bound calculation

Fraction bound = $\frac{Ion abudance of PL}{Ion abudance of pL + Ion abudance of free protein}$

Where PL represent protein-ligand

**Equation S3:** [PL] calculation

[PL] = Fraction bound × Protein Concentration (10 µM)

**Equation S4:** Free ligand calculation

[L] _free =_  [L] _total_ – [PL]
